# Wandsworth Infirmary

**Published:** 1903-05-16

**Authors:** 


### HOSPITAL ADMINISTRATION.

### CONSTRUCTION AND ECONOMICS.

### WANDSWORTH INFIRMARY.

If the ratepayers of the Borough of Wandsworth have any feeling for the unfortunates who go to their Union Infirmary when they are sick, they will insist on a rigorous investigation into the management of that institution. Within the last week or two there have been two cases which reflect on the intelligence and humanity of the staff. The first is the death, on April 20th, of a man named Michael Sadler. This man, formerly a commercial traveller, had fallen into intemperate habits, and when he entered the infirmary was suffering from alcoholic disease, which afterwards developed into delirium tremens. While in this condition he was restless and had to be put in a padded room, but seemed to be recovering, and was under no restraint at the time of his death. A post-mortem examination was held on his body, and Dr. Freyberger, who carried this out, stated indeed that the death was due to alcoholic disease, but that it had been hastened by violence which had resulted in two bruises and six broken ribs. Dr. Freyberger was of opinion, and Dr. Neale, the medical superintendent of the infirmary agreed with him, that the injury had been inflicted not more than eight and not less than five days before the man's death. Sadler had made no complaint of his treatment, and when his son, who visited him on Easter Sunday, asked him if he was well treated, Sadler had replied, "Yes, only they won't let me get out of bed." Presumably it was when he was being prevented from getting out of bed, that the blows which had bruised each side of his breast and broken his ribs had been inflicted. No attempt was made before the coroner to deny that violence had been inflicted, but it proved to be impossible to say who had been guilty of it. Six male "nurses" had had access to Sadler, but each of these denied using violence of any kind towards him, and there was no evidence to bring forward against anyone. That of an inmate of Wandsworth Workhouse, who had previously been in the infirmary, and who specified one of the nurses as having kicked him, was so evidently malicious that it could not be accepted as indicating the possible criminal. Yet a criminal there must be, though under the circumstances the coroner, Mr. Troutbeck, was compelled to advise the jury to return an open verdict. What we want to know—and what it is the duty of the guardians and the ratepayers of Wandsworth to find out is—whether the six men who are described as nurses, and one of whom in all probability struck the blows that hastened Sadler's death, have had any training whatever in the nursing of the sick? If not, it is the obvious duty of the people of Wandsworth to see that they are replaced as soon as possible by either men or women who have had such training, and who will be able to keep a restless patient in bed without breaking his ribs.

The date of this old man's death—April 20th—saw also the development of another tragedy connected with Wandsworth Infirmary. A young woman, named Lucy Nash, had given birth to a child there, and had afterwards passed with the child to the branch home in connection with it at Wandsworth Common. On the 20th of April—Monday—she left the home, and placed the baby out at nurse. Calling to see it on Wednesday, she was shocked at the change in its appearance; on Saturday it was worse, and she took it to the Infirmary, where it died on the Monday following. Of this case also Dr. Freyberger made a post-mortem examination, and found that the cause of death had been broncho-pneumonia, while the child had been suffering from commencing rickets and the effects of improper feeding. The broncho-pneumonia had not been recognised during the child's lifetime, nor had the rickets been diagnosed before the infant was taken out of the branch home, so that even if the woman who had the child to nurse was responsible for the improper feeding—and this was not shown—the union officials had failed to recognise two of the contributing causes of death. As Mr. Troutbeck said, the poor child had never been given a real chance at all.

In the infirmary there are also 168 children who, for lack of a children's hospital, sleep in the wards with the old people. Some of these are able to go out and play, but, although the Common is so near, these children have to be content with the balconies and airing courts for a playground. It has been suggested that the children might be taken out on the Common for walks under proper supervision, but this would mean an increase in the staff, and the infirmary committee also fear that the public would object to these convalescent pauper children having their airings there, especially as many of them are suffering from infectious diseases. Those so afflicted certainly should not go out among other people, but for other convalescents it seems hard not to be allowed out among the gorse and broom of the Common. Every resident has a right to the use of the Common—even a pauper child; it is not the preserve of the respectable folks who build handsome villas round it. But perhaps if the inhabitants of Wandsworth wake up they will be able to find a playground for these wards of theirs, or at least to provide them with a proper children's hospital.

